# Distribution, Function and Regulation of Type 6 Secretion Systems of Xanthomonadales

**DOI:** 10.3389/fmicb.2019.01635

**Published:** 2019-07-17

**Authors:** Ethel Bayer-Santos, Lucas de Moraes Ceseti, Chuck Shaker Farah, Cristina Elisa Alvarez-Martinez

**Affiliations:** ^1^Departamento de Microbiologia, Instituto de Ciências Biomédicas, Universidade de São Paulo, São Paulo, Brazil; ^2^Departamento de Genética, Evolução, Microbiologia e Imunologia, Instituto de Biologia, Universidade Estadual de Campinas, Campinas, Brazil; ^3^Departamento de Bioquímica, Instituto de Química, Universidade de São Paulo, São Paulo, Brazil

**Keywords:** Xanthomonadales, *Xanthomonas*, T6SS, bacterial killing, amoeba predation, effectors, toxins

## Abstract

Members of the Xanthomonadales order include several plant pathogens of significant economic and agricultural impact, such as *Xanthomonas* spp. Type 6 secretion systems (T6SSs) are contractile nanomachines used by many bacterial species to inject protein effectors into target prokaryotic and eukaryotic cells and provide a competitive advantage for bacteria in different environments. Effectors with antibacterial properties include peptidoglycan hydrolases, lipases and phospholipases that break down structural components of the cell envelope, promoting target-cell lysis; and RNases, DNAses, and NADases that affect target-cell metabolism, arresting growth. Effectors with anti-eukaryotic properties are functionally more diverse. The T6SS of *Xanthomonas citri* is the only example experimentally characterized so far within the Xanthomonadales order and displays anti-eukaryotic function by providing resistance to predation by amoeba. This T6SS is regulated at the transcriptional level by a signaling cascade involving a Ser/Thr kinase and an extracytoplasmic function (ECF) sigma factor. In this review, we performed *in silico* analyses of 35 genomes of Xanthomonadales and showed that T6SSs are widely distributed and phylogenetically classified into three major groups. *In silico* predictions identified a series of proteins with known toxic domains as putative T6SS effectors, suggesting that the T6SSs of Xanthomonadales display both anti-prokaryotic and anti-eukaryotic properties depending on the phylogenetic group and bacterial species.

## Introduction

The order Xanthomonadales includes many Gram-negative rod-shaped bacteria with very diverse physiological characteristics and habitats. Members of this group range from plant and human pathogens to non-pathogenic environmental bacteria that are able to survive in adverse conditions such as contaminated soil and hot springs (Saddler and Bradbury, 2005). Xanthomonadales isan early diverging branch of the Gammaproteobacteria (Williams et al., 2010). The taxonomy of the order is controversial, but a recent phylogenetic analysis has divided Xanthomonadales into two main branches comprising the families *Xanthomonadaceae* and *Rhodanobacteraceae* (Naushad et al., 2015). *Xanthomonadaceae* includes genera *Xanthomonas, Xylella, Stenotrophomonas, Pseudoxanthomonas, Luteimonas, Lysobacter, Thermomonas, Arenimonas*, and *Silanimonas*; while *Rhodanobacteraceae* includes genera *Rhodanobacter, Dyella, Frateuria, Luteibacter, Fulvimonas, Pseudofulvimonas, Aquimonas, Dokdonella*, and *Rudaea* (Naushad et al., 2015).

Species of the *Xanthomonadaceae* family have been by far the most studied due to their importance as plant pathogens. The genera *Xanthomonas* and *Xylella* contain species that promote disease in more than 400 economically important crops, including citrus, tomato, rice, cabbage, pepper, coffee, grapes, and olives (Leyns et al., 1984; Rapicavoli et al., 2018). Species within both genera vary in their ability to colonize different plant tissues and show a high degree of host specificity (Ryan et al., 2011). *Stenotrophomonas* is another important genus in *Xanthomonadaceae*. *Stenotrophomonas maltophilia* include several strains that are nosocomial pathogens, causing bacteremia, endocarditis and pneumonia in immunocompromised and cystic fibrosis patients (Adegoke et al., 2017). Conversely, species like *Stenotrophomonas rhizophila* are environmental bacteria found in association with plants and have a well-documented ability to promote plant growth, suppress colonization by plant pathogens and degrade a wide variety of xenobiotics, making them potential agents for biocontrol and bioremediation (Berg and Martinez, 2015). The genus *Lysobacter* comprises gliding predatory bacteria that display broad-spectrum lytic activity against nematodes, fungi, Gram-negative and Gram-positive bacteria (Christensen and Cook, 2009), including species of significant biotechnological and biocontrol interest (Ko et al., 2009; Hayward et al., 2010).

Availability of genomic data from an increasing number of Xanthomonadales species has provided important insights into environmental adaptations and physiological diversity. Gene clusters encoding bacterial secretion systems are recognized as key virulence factors of pathogenic species within the order (Büttner and Bonas, 2010). The type 6 secretion system (T6SS) is a molecular nanomachine that provides increased fitness to bacteria by firing a series of toxic effector proteins into neighbor competitor species, thus shaping bacterial communities. The T6SS of the biocontrol agent *Pseudomonas putida* kills phytopathogens upon co-infection *in planta*, and the *Agrobacterium tumefaciens* T6SS promotes plant colonization by providing a competitive advantage (Ma et al., 2014; Bernal et al., 2017). Anti-eukaryotic T6SSs are important for virulence in mammalian hosts, as well as for bacterial survival in the environment by providing resistance to predation by amoebas and exhibiting killing activity against fungi (Hachani et al., 2016; Bayer-Santos et al., 2018; Trunk et al., 2018). A role of T6SS in nutrient acquisition by the secretion of metal-scavenging proteins in the extracellular milieu has also been reported (Si et al., 2017a,b).

The sole T6SS representative of the Xanthomonadales order experimentally characterized to date from *Xanthomonas citri* pv. *citri* is required for resistance against predation by the soil amoeba *Dictyostelium discoideum* (Bayer-Santos et al., 2018), a yet unexplored aspect of xanthomonad biology that can be expected to be an important factor for environmental survival and dissemination. The secreted effectors and dynamics of *X. citri*-amoeba interactions are still elusive. The *X. citri* T6SS does not confer a competitive advantage in encounters with other Gram-negative bacteria and *X. citri* antibacterial activity is dependent on a type 4 secretion system (T4SS) (Souza et al., 2015; Bayer-Santos et al., 2018).

T6SSs are encoded in the genome of several species within Xanthomonadales. In this review, we performed *in silico* analyses of Xanthomonadales T6SSs to describe the distribution and genomic organization of T6SSs clusters in these species. Furthermore, we identified putative T6SS effectors that provided clues about the function of uncharacterized T6SS clusters in several Xanthomonadales species.

## Type 6 Secretion System

The T6SS is a contractile machinery composed of 13 core structural proteins. This system is evolutionarily related to the tail of bacteriophages (Basler and Mekalanos, 2012) and assembles into three major complexes: the *trans*-membrane complex, the baseplate and the tail. The *trans*-membrane complex is composed of three proteins TssM, TssL, and TssJ. The baseplate is formed by TssE, TssF, TssG, and TssK, and represents an adaptor between the *trans*-membrane complex and the tail. The tail has an internal tube formed by Hcp topped with VgrG and is enveloped by a contractile sheath composed of TssB and TssC (Nguyen et al., 2018). The assembly of the tail requires TssA, which interacts with baseplate, inner tube and sheath components and stabilizes the distal extremity of the tube (Planamente et al., 2016; Zoued et al., 2016; Dix et al., 2018). After contraction, the T6SS tail is recycled via the ATPase ClpV that disassembles the sheath into monomeric components (Kapitein et al., 2013). In addition to the core structural proteins described above, T6SS gene clusters also encode accessory proteins, which comprise components required for the assembly of the secretion apparatus, regulatory subunits acting transcriptionally or post-translationally to control the expression or the activity of the T6SS, and effectors and immunity proteins required for its function (Silverman et al., 2012).

T6SSs deliver protein effectors into diverse cell types including prokaryotic and eukaryotic cells in a contact-dependent manner (Cianfanelli et al., 2016; Hachani et al., 2016). T6SSs were also reported to display contact-independent functions in which secreted effectors facilitate the acquisition of nutrients (Wang et al., 2015; Si et al., 2017a,b). T6SS gene clusters have been classified into four subtypes (T6SS^i-iv^) (Boyer et al., 2009; Bröms et al., 2010; Russell et al., 2014; Bock et al., 2017): (i) the majority of T6SSs belong to subtype T6SS^i^ and are present in Proteobacteria; (ii) the *Francisella* pathogenicity island-like systems were classified as T6SS^ii^ (Bröms et al., 2010); (iii) Bacteroidetes T6SSs are distinct from the first two and were classified as T6SS^iii^ (Russell et al., 2014); and (iv) a contractile system from *Amoebophilus asiaticus* was classified T6SS^iv^ (Bock et al., 2017). Proteobacteria T6SS^i^ are the most diverse and have been further subdivided into five phylogenetic clades (Boyer et al., 2009). Xanthomonadales harbor three subtypes of T6SS^i^ belonging to clades 1, 3, and 4, which will be discussed below.

## Genomic Architecture of Xanthomonadales T6SS Clusters

From 71 Xanthomonadales species genomes retrieved from the KEGG database (Kanehisa and Goto, 2000), we identified 35 genomes harboring one or two T6SS clusters (Supplementary Table S1). Distribution of T6SS does not show a clear correlation with species lifestyles and they are found in several environmental bacteria and phytopathogenic species that colonize distinct plant tissues (vascular and non-vascular pathogens) (Supplementary Table S1). T6SS clusters are absent in xylem-limited phytopathogens with reduced genomes, including *X. albilineans* (3.78 Mb) and all members of the *Xylella* genus (2.5 Mb).

**FIGURE 1:**
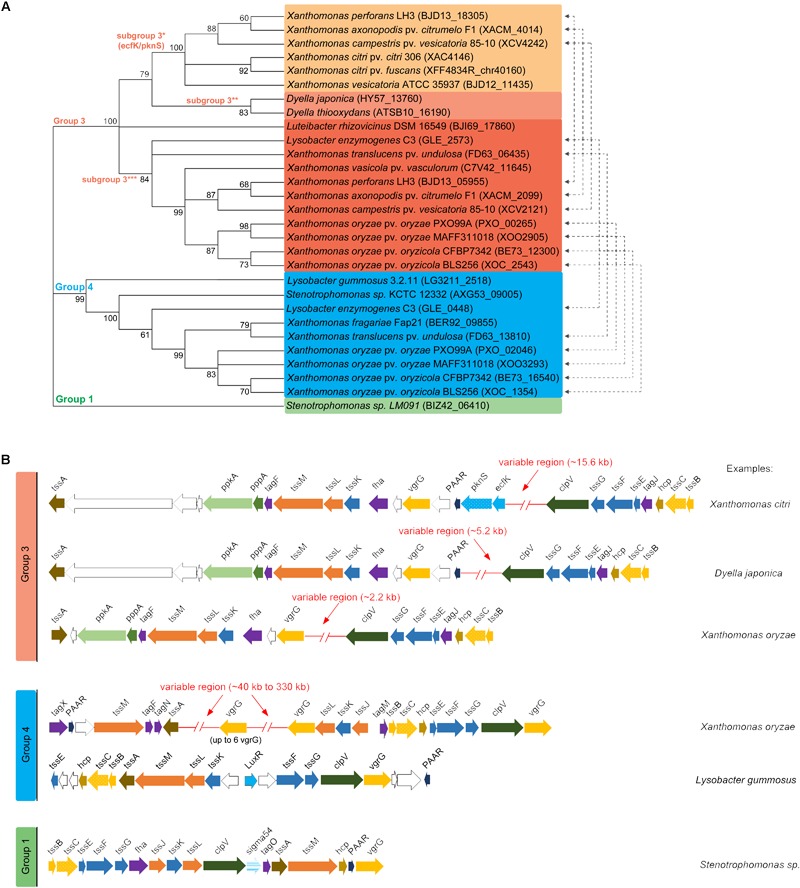
Phylogenetic classification of T6SSs within Xanthomonadales and genomic organization of T6SS gene clusters. **(A)** Phylogenetic distribution of Xanthomonadales T6SS clusters. Maximum-likelihood consensus tree with 1000 bootstrap replicates built with amino acid sequence of TssC (XAC4146) homologs aligned and grouped using MEGA 7.0 (Kumar et al., 2016). **(B)** Schematic representation of genomic regions encoding the different subtypes of T6SSs.

According to phylogenetic analyses using the sheath component TssC, T6SSs clusters separate into three groups matching clades/groups 1, 3, and 4 proposed by Boyer et al. (2009) (Figure 1A). Similarly, analysis of T6SS distribution in plant-associated bacteria that included several members of the *Xanthomonas* genus has shown an overrepresentation of these three clades (Bernal et al., 2018). Group 3 presents the most heterogeneous distribution among plant-associated bacteria and the Xanthomonadales representatives are clustered in a clade that includes *Burkholderia* species (Bernal et al., 2018). Xanthomonadales group 4 T6SSs belong to subgroup 4B2 described by Bernal et al. (2018), which also includes *Ralstonia* and *Burkholderia* species. The sole member of group 1 is found in a *Stenotrophomonas* sp. isolated from the phyllosphere and a phylogenetic analysis showed that it belongs to the subclade 1.2A described by Bernal et al. (2018), which includes *P. putida* species. Each group displays a unique genetic architecture, contains different T6SS-associated genes (Tag proteins) and present variable regions within or in the vicinity of the structural gene clusters, which contain putative effectors and/or regulatory proteins (Figure 1B). The characteristics of each group are further described in detail below.

Group 3 presents two main clusters of structural genes separated by an insertion in which the content varies depending on the bacterial species, ranging from ∼2.2 to 15.6 kb (Figure 1B). Group 3 was further divided into three subclades (hereafter referred to as subgroup 3^∗^, 3^∗∗^, and 3^∗∗∗^) (Figure 1A,B). Division of *Xanthomonas* group 3 T6SSs in two subclades is also observed in the phylogenetic analysis by Bernal et al. (2018), which did not include *Dyella* species. Group 3^∗^ contains the only Xanthomonadales T6SS functionally characterized to date from *X. citri* (Figure 1A) (Bayer-Santos et al., 2018) and is restricted to *Xanthomonas* species, a few of them also containing a second T6SS from subgroup 3^∗∗∗^ (Figure 1). The presence of one representative from each subgroup in some species may indicate distinct functions in bacterial physiology, despite similarities in cluster organization.

Group 3 shows unique features such as the presence of components from the PpkA-PppA-FHA post-translational phosphorylation pathway (Mougous et al., 2007) and its repressor TagF (Silverman et al., 2011; Lin et al., 2018). It also contains TagJ, which interacts with the ATPase ClpV and the sheath component TssB to control the disassembly of the contracted sheath (Forster et al., 2014). TagJ co-evolved with a specific subset of the TssB/TssC/ClpV gene cluster (Forster et al., 2014). The TssA component from group 3 is closely related to homologs from group 4 T6SSs, belonging to the same phylogenetic clade 1 (TssA1) (Dix et al., 2018), while TssA from group 1 T6SS shows a divergent C-terminal region and belongs to clade 2 (Dix et al., 2018). In *X. citri*, the variable region is ∼15.6 kb and contains the extracytoplasmic function (ECF) sigma factor EcfK and its cognate kinase PknS, which were shown to be required for T6SS activation and function (Bayer-Santos et al., 2018). Curiously, EcfK/PknS are not present in the most similar T6SS cluster from *Dyella japonica* and *Dyella thiooxydans* (Figure 1A), which has an intermediate-size variable region of ∼5.2 kb. Despite the differences in the variable region, T6SS from *D. japonica* and *D. thiooxydans* are very similar to *X. citri* T6SS and present conserved genes of unknown function downstream of the *PAAR* and *vgrG* genes (Figure 1B, white arrows). A third genetic architecture within group 3 can be observed in a branch comprising *Xanthomonas oryzae* pv. *oryzae, Xanthomonas oryzae* pv. *oryzicola*, and *Lysobacter enzymogenes* (subgroup 3^∗∗∗^). These species contain even smaller insertions in the variable region (∼2.2 kb) and *tssA* appears in a different orientation (Figure 1B). Bacterial species carrying this subtype of T6SS usually have a second T6SS belonging to group 4 or subgroup 3^∗^ (Figure 1A, blue).

T6SSs classified as group 4 display two genetic architectures. In the majority of species, the structural core genes are organized in conserved clusters and variability is found in the vicinity of *vgrG* genes. These variable regions are very large – ranging from 40 kb to 75 kb – and contain several duplications of *vgrG* (up to 6), proteins with domains of unknown functions (DUFs) and proteins with putative toxic domains (Table 1). In some species, such as *Xanthomonas fragariae*, the cluster of structural genes composed of *tssM, tagF, tagN*, and *tssA* are located ∼300 kb from the other structural cluster composed of *tssJ, tssK, tssL, vgrG*. Another interesting and unique feature of T6SSs from group 4 is the presence of a gene encoding TagX, which is a membrane-associated peptidoglycan hydrolase proposed to help degrade the bacterial wall for the insertion of T6SS machinery (Weber et al., 2016). Group 4 T6SSs also contain the associated genes *tagN* and *tagM*, but their role in T6SS biogenesis or regulation was not yet clarified. *Lysobacter gummosus* is the only example belonging to group 4 that contains a divergent version of T6SS, displaying a different genetic architecture (Figure 1B).

**Table 1:** List of PAAR and VgrG proteins from Xanthomonadales and putative toxic effectors identified by Bastion6 software.

Species	T6SS Group	PAAR	Orphan PAAR	VgrG (class)	Orphan VgrG (class)	Bastion6 predicted T6SS effectors	
						*N*	Description^1,2^
*X. vesicatoria* ATCC 35937	3^∗^	1	0	1 (I)	0	4	Hypothetical

*X. vasicola* pv. *vasculorum* SAM119	3^∗∗∗^	0	0	1 (I)	0	1	Hypothetical

*X. translucens* pv. *undulosa* Xtu 4699	3^∗∗∗^	0	2	1 (I)	0	1	Hypothetical
						
	4	1		2 (II)		12^#^	Tle1-like, DUF3304 (2), neuraminidase
						
						2 orphans	Tox-REase-5 (2)

*X. perforans* LH3	3^∗^	1	0	1 (I)	0	3	Acid phosphatase, EEP endo/exonuclease/phosphatase
						
	3^∗∗∗^	0		1 (I)		2	TIR_2 superfamily

*X. oryzae* pv. *oryzicola* CFBP7342	3^∗∗∗^	0	3	1 (I) and 1 (II)	1 (I) and 10 (II)	3	Hypothetical
						
	4	1		4 (II)		19	Tle1-like (4), DUF3304 (5), hydrolase
						
						55 orphans	Tle1-like (2), Tle3-like (2), Tle4-like (2), DUF2875 (5), DUF3304 (4), DUF1800, DUF1501, catalase, peptidase (2), PAAR/Rhs MafB19-deaminase, haemolysin, Tox-REase-5

*X. oryzae* pv. *oryzicola* BLS256	3^∗∗∗^	0	1	2 (I) and 1 (II)	3 (I) and 5 (II)	1	Hypothetical
						
	4	1		2 (I) and 3 (II)		14	Tle1-like (4), DUF3304 (3), hydrolase
						
						39 orphans	Tle1-like (2), Tle3-like (4), Tle4-like (3), DUF2875 (5), DUF3304 (5), DUF493, peptidase, transglycosylase, carboxypeptidase, Rhs repeat proteins (2), oxidoreductase

*X. oryzae* pv. *oryzae* PXO99A	3^∗∗∗^	0	5	1 (I) and 1 (II)	1 (I) and 5 (II)	2	Hypothetical
						
	4	1		3 (II)		12	Tle1-like (2), DUF3304 (2), transglycosylase
						
						13 orphans	Tle3-like (5), DUF2875 (4)

*X. oryzae* pv. *oryzae* MAFF 311018	3^∗∗∗^	0	3	1 (I)	2 (I) and 7 (II)	0	
						
	4	1		5 (II)		14	Tle1-like (2), DUF3304 (5), DUF2345
						
						51 orphans	Tle1-like (4), DUF3304 (6), Tle3-like (5), DUF2875 (5), murein hydrolase D, muraminidase (2), carboxypeptidase, peptidase, PAAR/Rhs XOO_2897-like deaminase, Tox-REase-5 (2)

*X. fragariae* Fap21	4	1	0	4 (II)	2 (I) and 4 (II)	12	Hypothetical
						
						27 orphans	Tle1-like (2), DUF3304 (6), glycoside hydrolase, Rhs repeat protein, PAAR/DUF4150 Colicin-DNAse

*X. citri* pv. *fuscans*	3^∗^	1	0	1 (I)	0	4	Acid phosphatase, EEP endo/exonuclease/phosphatase

*X. citri* pv. *citri* 306	3^∗^	1	0	1 (I)	0	4	Acid phosphatase, EEP endo/exonuclease/phosphatase, adhesin

*X. campestris* pv. *vesicatoria* 85-10	3^∗^	1	0	1 (I)	0	4	Acid phosphatase, EEP endo/exonuclease/phosphatase
						
	3^∗∗∗^	0		1 (I)		4	TIR_2 superfamily

*X. axonopodis* pv. *citrumelo* F1	3^∗^	1	0	1 (I)	0	4	Acid phosphatase, EEP endo/exonuclease/phosphatase
						
	3^∗∗∗^	0		1 (I)		3	TIR_2 superfamily

*Stenotrophomonas* sp. LM091	1	1	0	1 (II)	0	5	Tle3-like (2), DUF4274, DUF2875

*Stenotrophomonas* sp. KCTC 12332	4	1	2	1 (I) and 1 (II)	1 (II)	4	Hypothetical
						
						6 orphans	Tle1-like, Tle4-like, DUF4287

*L. rhizovicinus* DSM 16549	3^∗∗∗^	0	0	1 (I)	0	1	Hypothetical

*L. gummosus* 3.2.11	4	1	2	1 (I)	1 (I)	4	Transglycosylase, peptidoglycan-binding protein
						
						7 orphans	DUF4157, peptidase, DUF3244/DUF3218

*L. enzymogenes* C3	3^∗∗∗^	0	3	1 (I)	1 (I)	0	
						
	4	0		3 (II)		7	Tle4-like protein, hydrolase, peptidase, phospholipase
						
						3 orphans	GTP-binding protein, TPR-repeat protein

*D. thyooxidans* ATSB10	3^∗∗^	1	0	1 (I)	0	1	Hypothetical

*D. japonica* A8	3^∗∗^	1	0	1 (I)	2 (II)	2	DUF5636, amidase
						
						7 orphans	Phospholipase, muraminidase, peptidase,TPR repeat proteins (3)

*^#^All genes flanking the two VgrGs were analyzed. ^*1*^Only proteins with putative toxic domains or DUFs previously associated with T6SS are described. ^*2*^Number of proteins with identical domains are indicated in brackets when >1.*

The only example of a Group 1 T6SS is found in *Stenotrophomonas* sp. LM91. This system is very similar to the T6SS from *Vibrio cholerae* and contains the sigma factor σ^54^ transcriptional regulator, which was reported to control the expression of T6SS in other bacteria (Bernard et al., 2011). Group 1 T6SS also contains a gene encoding the associated protein TagO, but its function is still unknown.

## Function of Putative Xanthomonadales T6SS Effectors

T6SSs translocate effectors by decorating the Hcp-VgrG-PAAR puncturing device that is propelled against target cells, thus delivering a cocktail of effectors after each contraction event. The current model suggests that effectors can either interact with one of these three proteins, named “cargo” effectors, or be presented as an extra domain within the same proteins, named “specialized” effectors (Durand et al., 2014; Cianfanelli et al., 2016).

In order to assess the repertoire of Xanthomonadales T6SSs effectors, we manually analyzed the genomic regions encoding the structural components Hcp, VgrG, and PAAR to search for specialized effectors. All Xanthomonadales species analyzed display only one copy of Hcp that is associated with a T6SS structural cluster, and these Hcps do not present a C-terminal extension (Supplementary Table S2A). The VgrG repertoire of Xanthomonadales seems to be more diverse. VgrGs are categorized into three classes (De Maayer et al., 2011): the first is composed of proteins with a N-terminal VgrG domain; the second class is formed by VgrG proteins carrying a C-terminal domain of unknown function DUF2345, which is required for interaction with cargo effectors (Flaugnatti et al., 2016); and the third class comprises the evolved- or specialized-VgrG that carry a C-terminal toxic domain. In Xanthomonadales, class I VgrGs seem to be predominantly associated with the T6SSs belonging to group 3 (Table 1 and Supplementary Table S2B). Class II VgrGs are more abundant in species that carry a T6SS belonging to group 4 (Table 1 and Supplementary Table S2B). In addition, Xanthomonadales species that contain T6SS clusters from group 4 usually have orphan VgrGs scattered in the genome (Supplementary Table S2B), while species with T6SSs from subgroup 3^∗^ do not harbor orphan VgrGs. Interestingly, a high number of orphan VgrGs are found in the genomes of *X. oryzae* from different strains. Moreover, no bona fide specialized VgrGs (class III) were detected in the analyzed genomes (Table 1 and Supplementary Table S2B). PAAR proteins were associated with T6SS structural gene clusters in all groups, except species from subgroup 3^∗∗∗^ (Figure 1, Table 1, and Supplementary Table S2C). Most species harboring a subgroup 3^∗∗∗^ T6SS that lack a PAAR protein also encode an additional T6SS cluster in their genomes, either from subgroup 3^∗^ or 4 (Figure 1A). At this point, it is unclear whether PAAR-like proteins could be shared between two systems or whether subgroup 3^∗∗∗^ systems are non-functional due to the loss of an associated PAAR protein. Nevertheless, none of the Xanthomonadales PAAR proteins associated with T6SS clusters have extended domains coding for putative toxic proteins (Supplementary Table S2C). Orphan PAAR proteins are present in genomes that contain a group 4 T6SS cluster, some of them with extended sizes that might encode effector functions (Supplementary Table S2C). Among them, three proteins that belong to the modular PAAR-Rhs-toxin group of antibacterial toxins (Ma et al., 2017) are present in the genomes of *X. fragarie, X. oryzae* pv. *oryzae*, and *X. oryzae* pv. *oryzicola* (Supplementary Tables S2C, S3).

To search for cargo effectors using *in silico* analyses, we arbitrarily chose to analyze a fixed number of 10 genes immediately flanking all T6SS VgrGs using Bastion6 software (Wang et al., 2018). We also searched for genes encoding DUF4123, DUF2169 or DUF1795-containing proteins, which act as adaptors for effector recruitment by T6SSs (Liang et al., 2015; Unterweger et al., 2015; Bondage et al., 2016). Genes encoding DUF1795 are not present in Xanthomonadales genomes, while DUF2169 genes are located in association with VgrG (Supplementary Table S2D), as previously described (Liang et al., 2015; Unterweger et al., 2015). Fourteen DUF4123-containing proteins were found encoded in Xanthomonadales genomes, not associated with structural clusters or orphan VgrGs (Supplementary Table S2E). We retrieved the two genes located immediately downstream from each DUF4123-encoding gene for effector prediction using Bastion6. Antibacterial cargo effectors are usually encoded in bicistronic units with genes encoding cognate immunity proteins, targeting components of the bacterial cell-envelope and/or nucleic acids, such as peptidoglycan hydrolases, amidases, lipases, and nucleases (Lien and Lai, 2017). Effectors with anti-eukaryotic properties are less studied and functionally more diverse and include proteins involved in actin-crosslinking, lipases, deaminases and catalases (Jiang et al., 2014; Lien and Lai, 2017).

Analysis of the set of predicted T6SS effectors from Xanthomonadales showed a high number of putative antibacterial toxins associated with group 4 T6SS, which display hydrolase, lipase, carboxypeptidase and muraminidase domains (Table 1 and Supplementary Table S3). Interestingly, most genomes harbouring a group 4 T6SS present multiple copies of members of the superfamily of antibacterial T6SS lipase effectors (Tle), more specifically from families Tle1, Tle3, and Tle 4 (Russell et al., 2013) (Table 1 and Supplementary Table S3). Furthermore, Tle1 copies are found in association with T6SS clusters from group 4 in most genomes (Table 1 and Supplementary Table S3). Tle1 homologs from *Burkholdeia thailandensis* and *Escherichia coli* EAEC Sci-1 T6SS were shown to have antibacterial activity (Russell et al., 2013; Flaugnatti et al., 2016). In *X. fragariae*, putative antibacterial toxins containing hydrolase domains and a colicin-DNAse domain are associated with orphan VgrGs (Table 1 and Supplementary Table S3). Similarly, a variety of putative effectors with hydrolase and phospholipase domains were found associated to the variable regions of *L. enzymogenes* group 4 cluster, which has 3 class II *vgrG* genes (Table 1 and Supplementary Table S3). Interestingly, five putative predicted effectors belonging to the Tox-REase-5 family of restriction endonucleases that includes TseT, a previously described antibacterial toxin from *Pseudomonas aeruginosa* H2-T6SS (Burkinshaw et al., 2018) are present in association with orphan DUF4123-containing proteins in *X. oryzae* strains and *Xanthomonas translucens* pv. *undulosa* (Table 1). As no example belonging to Xanthomonadales group 4 T6SS has been functionally characterized to date, based on the domains of putative effectors secreted by these systems, we hypothesize that members of group 4 might display antibacterial activity rather than anti-eukaryotic activity as observed for the *X. citri* T6SS from group 3^∗^ (Bayer-Santos et al., 2018). Interestingly, group 4 T6SSs are mostly found in Xanthomonadales genomes that lack the antibacterial T4SS (Souza et al., 2015; Sgro et al., 2019), as exemplified by all *X. oryzae* strains.

Prediction of T6SS effectors associated with VgrGs from group 3 identified a limited number of proteins of unknown function (Table 1 and Supplementary Table S3). For the T6SSs from subgroup 3^∗^, which are homologous to the anti-amoeba T6SS from *X. citri*, two proteins were frequently identified in the vicinity of VgrGs: an acid phosphatase and a protein with lectin and phosphodiesterase domains from the EEP family (endo/exonuclease/phosphatase) (Table 1). A distinct set of conserved hypothetical proteins were identified in subgroup 3^∗∗∗^, including some with a TIR_2 domain typical of Toll-like receptors. Bacterial proteins containing these domains have been originally implicated in virulence by subversion of host immune responses, but recent work showed their roles as NADases that cleave NAD^+^ and interfere with cellular metabolism (Cirl et al., 2008; Essuman et al., 2018). On the other hand, *D. japonica* group 3 T6SS is the sole representative in the group that presents several predicted antibacterial T6SS effectors (proteins containing amidase, muraminidase, and phospholipase domains) in the vicinity of VgrG, suggesting a role as an anti-prokaryotic weapon (Table 1). These observations lead us to speculate that distinct subgroups within group 3 might display diverse functions in Xanthomonadales.

## Regulation of T6SSS

Regulation of T6SSs assembly and firing events occur at different levels: transcriptional, posttranscriptional, and post-translational. Transcriptional regulation of T6SS genes is highly variable among species and several components have been implicated in this activity, including the nucleoid-structuring protein H-NS, σ^54^ and regulators of nutrient acquisition pathways such as Fur and PhoB-PhoR (Brunet et al., 2011, 2015; Silverman et al., 2012). The *X. citri* T6SS is regulated at the transcriptional level by a mechanism involving an alternative sigma factor of the ECF family named EcfK and a transmembrane eukaryotic-like serine-threonine kinase (PknS), which is required for activation of EcfK (Bayer-Santos et al., 2018).

Interestingly, T6SS clusters from subgroup 3^∗∗∗^ that do not contain EcfK/PknS carry a gene for a LysR-type transcriptional regulator that is predicted to be co-transcribed with the *tssA* gene (Figure 1 and Supplementary Table S4). Genes encoding a VirA/VirG-like two-component system are also found in a putative operon associated with *lysR-tssA* in all subgroup 3^∗∗∗^ T6SS, except for *Luteibacter rhizovicinus* that form a distinct branch within the group (Figure 1 and Supplementary Table S4). This conservation in genome organization and the absence of *ecfK/pknS* suggest that these regulators might be involved in the control of subgroup 3^∗∗∗^ T6SS gene expression. T6SS from *Dyella* spp. form a distinct branch in group 3 and do not present *ecfK/pknS* homologs or *lysR*-type genes near their T6SS clusters. No conserved gene encoding a transcriptional regulator was identified in T6SS clusters from group 4, except for a LuxR homolog that is present in the divergent *L. gummosus* cluster.

Distribution of T6SS putative post-translational regulators also differ among distinct clades. T6SS clusters from group 3 contain *tagF-pppA-ppkA-fha* genes, suggesting that activation of T6SS assembly and firing is dependent on the activation of the kinase PpkA, as originally described in *P. aeruginosa* (Mougous et al., 2007; Casabona et al., 2013). Recent work has demonstrated that PpkA acts by counteracting the inhibitory effect of TagF on T6SS activity, and the distinction between defensive and offensive T6SSs is mainly determined by the upstream input from protein(s) responsible for activation of PpkA (Lin et al., 2018; Ostrowski et al., 2018). In the defensive T6SS of *P. aeruginosa*, an incoming attack is sensed in the periplasm by TagQRST, while in the offensive *Serratia marcensens* T6SS, the signal is sensed by RtkS (Ostrowski et al., 2018). The offensive/defensive model has been described in only a few antibacterial T6SS and the mechanism of post-translational activation of anti-eukaryotic machines is possibly different.

Whether the post-translational regulatory cascade depending on tagF-pppA-ppkA-fha genes are functional in Xanthomonadales remains to be determined experimentally. Interestingly, T6SS clusters from group 4 do not encode *ppkA* or *pppA*, but instead contain a *tagF* gene (Figure 1), suggesting that a yet undescribed mechanism may be involved in relieving repression imposed by TagF in this group. The group 1 T6SS clusters from *Stenotrophomonas* sp. do not encode any component of the post-translational regulatory pathway, suggesting that activation occurs at the transcriptional level through σ^54^.

## Perspectives

Studies on the function, regulation and characterization of effector repertoire of T6SSs from Xanthomonadales is only just beginning. Much work is still required to understand how these systems contribute to the biology and pathogenesis of members of these important groups of bacteria. Further experimental evidence is needed to clarify whether the different subtypes of T6SSs pointed out in this review function as antibacterial and/or anti-eukaryotic weapons and whether the predicted putative effectors identified here are bona fide cargo proteins.

## Author Contributions

EB-S and CA-M performed *in silico* analysis, wrote and edited the review. LC performed *in silico* analysis and wrote the review. CF wrote and edited the review.

## Conflict of Interest Statement

The authors declare that the research was conducted in the absence of any commercial or financial relationships that could be construed as a potential conflict of interest.

## References

[B1] Adegoke A. A. Stenström T. A. Okoh A. I. (2017). *Stenotrophomonas maltophilia* as an emerging ubiquitous pathogen: looking beyond contemporary antibiotic therapy. *Front. Microbiol.* 8:2276. 10.3389/fmicb.2017.02276 29250041 PMC5714879

[B2] Basler M. Mekalanos J. (2012). Type 6 secretion dynamics within and between bacterial cells. *Science* 337:815. 10.1126/science.1222901 22767897 PMC3557511

[B3] Bayer-Santos E. Lidia dos Passos L. de Moraes Ceseti L. Ratagami C. Y. de Santana E. S. da Silva A. M. (2018). Xanthomonas citri T6SS mediates resistance to *Dictyostelium* predation and is regulated by an ECF σ factor and cognate Ser/Thr kinase. *Environ. Microbiol.* 20 1562–1575. 10.1111/1462-2920.14085 29488354

[B4] Berg G. Martinez J. L. (2015). Friends or foes: can we make a distinction between beneficial and harmful strains of the *Stenotrophomonas maltophilia* complex? *Front. Microbiol.* 6:241 10.3389/fmicb.2015.00241 PMC4379930 25873912

[B5] Bernal P. Allsopp L. P. Filloux A. Llamas M. A. (2017). The *Pseudomonas putida* T6SS is a plant warden against phytopathogens. *ISME J.* 11 972–987. 10.1038/ismej.2016.169 28045455 PMC5363822

[B6] Bernal P. Llamas M. A. Filloux A. (2018). Type VI secretion systems in plant-associated bacteria. *Environ. Microbiol.* 20 1–15. 10.1111/1462-2920.13956 29027348 PMC5813230

[B7] Bernard C. S. Brunet Y. R. Gavioli M. Lloubès R. Cascales E. (2011). Regulation of type VI secretion gene clusters by σ^54^ and cognate enhancer binding proteins. *J Bacteriol.* 193 2158–2167. 10.1128/JB.00029-11. 21378190 PMC3133059

[B8] Bock D. Medeiros J. Tsao H. Penz T. Weiss G. Aistleitner K. (2017). *In situ* architecture, function, and evolution of a contractile injection system. *Science* 357 713–717. 10.1126/science.aan7904 28818949 PMC6485382

[B9] Bondage D. D. Lin J. Ma L. Kuo C. Lai E. (2016). VgrG C terminus confers the type VI effector transport specificity and is required for binding with PAAR and adaptor – effector complex. *Proc. Natl. Acad. Sci. U.S.A.* 113 E3931–E3940. 10.1073/pnas.1600428113 27313214 PMC4941472

[B10] Boyer F. Fichant G. Berthod J. Vandenbrouck Y. Attree I. (2009). Dissecting the bacterial type VI secretion system by a genome wide *in silico* analysis: what can be learned from available microbial genomic resources? *BMC Genomics* 10:104. 10.1186/1471-2164-10-104 19284603 PMC2660368

[B11] Bröms J. E. Sjöstedt A. Lavander M. (2010). The role of the *Francisella tularensis* pathogenicity island in type VI secretion, intracellular survival, and modulation of host cell signaling. *Front. Microbiol.* 1:136. 10.3389/fmicb.2010.00136 21687753 PMC3109350

[B12] Brunet Y. R. Bernard C. S. Gavioli M. Lloubès R. Cascales E. (2011). An epigenetic switch involving overlapping fur and DNA methylation optimizes expression of a type VI secretion gene cluster. *PLoS Genet.* 7:e1002205. 10.1371/journal.pgen.1002205 21829382 PMC3145626

[B13] Brunet Y. R. Khodr A. Logger L. Aussel L. Mignot T. Rimsky S. (2015). H-NS silencing of the *salmonella* pathogenicity island 6-encoded type VI secretion system limits *Salmonella enterica* serovar typhimurium interbacterial killing. *Infect. Immun.* 83 2738–2750. 10.1128/IAI.00198-15 25916986 PMC4468533

[B14] Burkinshaw B. J. Liang X. Wong M. Le A. N. H. Lam L. Dong T. G. (2018). A type VI secretion system effector delivery mechanism dependent on PAAR and a chaperone–co-chaperone complex. *Nat. Microbiol.* 3 632–640. 10.1038/s41564-018-0144-4. 29632369

[B15] Büttner D. Bonas U. (2010). Regulation and secretion of *Xanthomonas* virulence factors. *FEMS Microbiol. Rev.* 34 107–133. 10.1111/j.1574-6976.2009.00192.x 19925633

[B16] Casabona M. G. Silverman J. M. Sall K. M. Boyer F. Couté Y. Poirel J. (2013). An ABC transporter and an outer membrane lipoprotein participate in posttranslational activation of type VI secretion in *Pseudomonas aeruginosa*. *Environ. Microbiol.* 15 471–486. 10.1111/j.1462-2920.2012.02816.x 22765374 PMC3467343

[B17] Christensen P. Cook F. D. (2009). *Lysobacter*, a new genus of nonfruiting. gliding bacteria with a high base ratio. *Int. J. Syst. Bacteriol.* 28 367–393. 10.1099/00207713-28-3-367

[B18] Cianfanelli F. R. Monlezun L. Coulthurst S. J. (2016). Aim. load, fire: the type VI secretion system, a bacterial nanoweapon. *Trends Microbiol.* 24 51–62. 10.1016/j.tim.2015.10.005 26549582

[B19] Cirl C. Wieser A. Yadav M. Duerr S. Schubert S. Fischer H. (2008). Subversion of toll-like receptor signaling by a unique family of bacterial Toll/interleukin-1 receptor domain-containing proteins. *Nat. Med.* 14:399. 10.1038/nm1734 18327267

[B20] De Maayer P. Venter S. Kamber T. Duffy B. Coutinho T. Smits T. (2011). Comparative genomics of the Type VI secretion systems of *Pantoea* and *Erwinia* species reveals the presence of putative effector islands that may be translocated by the VgrG and Hcp proteins. *BMC Genomics* 12:576. 10.1186/1471-2164-12-576 22115407 PMC3235180

[B21] Dix S. R. Owen H. J. Sun R. Ahmad A. Shastri S. Spiewak H. L. (2018). Structural insights into the function of type VI secretion system TssA subunits. *Nat. Commun.* 9:4765. 10.1038/s41467-018-07247-1 30420757 PMC6232143

[B22] Durand E. Cambillau C. Cascales E. Journet L. (2014). VgrG. Tae, Tle, and beyond: the versatile arsenal of Type VI secretion effectors. *Trends Microbiol.* 22 498–507. 10.1016/j.tim.2014.06.004 25042941

[B23] Essuman K. Summers D. Sasaki Y. Mao X. Yim A. DiAntonio A. (2018). TIR domain proteins are an ancient family of NAD(+)-consuming enzymes. *Curr. Biol.* 28 421–430. 10.1016/j.cub.2017.12.024 29395922 PMC5802418

[B24] Flaugnatti N. Le T. Canaan S. Aschtgen M. Nguyen V. Blangy S. (2016). A phospholipase A1 antibacterial type VI secretion effector interacts directly with the C-terminal domain of the VgrG spike protein for delivery. *Mol. Microbiol.* 99 1099–1118. 10.1111/mmi.13292 26714038

[B25] Forster A. Planamente S. Manoli E. Lossi N. S. Freemont P. S. Filloux A. (2014). Coevolution of the ATPase ClpV, the sheath proteins TssB and TssC and the accessory protein TagJ/HsiE1 distinguishes type VI secretion classes. *J. Biol. Chem.* 289 33032–33043. 10.1074/jbc.M114.600510 25305017 PMC4239648

[B26] Hachani A. Wood T. E. Filloux A. (2016). Type VI secretion and anti-host effectors. *Curr. Opin. Microbiol.* 29 81–93. 10.1016/j.mib.2015.11.006 26722980

[B27] Hayward A. C. Fegan N. Fegan M. Stirling G. R. (2010). *Stenotrophomonas* and *Lysobacter*: ubiquitous plant-associated gamma-*proteobacteria* of developing significance in applied microbiology. *J. Appl. Microbiol.* 108 756–770. 10.1111/j.1365-2672.2009.04471.x 19702860

[B28] Jiang F. Waterfield N. R. Yang J. Yang G. Jin Q. (2014). A *Pseudomonas aeruginosa* type VI secretion phospholipase D effector targets both prokaryotic and eukaryotic cells. *Cell Host Microbe* 15 600–610. 10.1016/j.chom.2014.04.010 24832454

[B29] Kanehisa M. Goto S. (2000). KEGG: kyoto encyclopedia of genes and genomes. *Nucleic Acids Res.* 28 27–30. 10.1093/nar/28.1.27 10592173 PMC102409

[B30] Kapitein N. Bönemann G. Pietrosiuk A. Seyffer F. Hausser I. Locker J. K. (2013). ClpV recycles VipA/VipB tubules and prevents non-productive tubule formation to ensure efficient type VI protein secretion. *Mol. Microbiol.* 87 1013–1028. 10.1111/mmi.12147 23289512

[B31] Ko H. S. Jin R. De Krishnan H. B. Lee S. B. Kim K. Y. (2009). Biocontrol ability of *Lysobacter antibioticus* HS124 against phytophthora blight is mediated by the production of 4-hydroxyphenylacetic acid and several lytic enzymes. *Curr. Microbiol.* 59 608–615. 10.1007/s00284-009-9481-0 19727949

[B32] Kumar S. Stecher G. Tamura K. (2016). MEGA7: molecular evolutionary genetics analysis version 7.0 for bigger datasets. *Mol. Biol. Evol.* 33 1870–1874. 10.1093/molbev/msw054 27004904 PMC8210823

[B33] Leyns F. De Cleene M. Swings J. G. De Ley J. (1984). The host range of the genus *Xanthomonas*. *Bot. Rev.* 5 308–356. 10.1007/BF02862635

[B34] Liang X. Moore R. Wilton M. Wong M. J. Q. Lam L. Dong T. G. (2015). Identification of divergent type VI secretion effectors using a conserved chaperone domain. *Proc. Natl. Acad. Sci. U.S.A.* 112 9106–9111. 10.1073/pnas.1505317112 26150500 PMC4517263

[B35] Lien Y.-W. Lai E.-M. (2017). Type VI secretion effectors: methodologies and biology. *Front. Cell. Infect. Microbiol.* 7:254. 10.3389/fcimb.2017.00254 28664151 PMC5471719

[B36] Lin J. S. Pissaridou P. Wu H. H. Tsai M. D. Filloux A. Lai E. M. (2018). TagF-mediated repression of bacterial type VI secretion systems involves a direct interaction with the cytoplasmic protein Fha. *J. Biol. Chem.* 293 8829–8842. 10.1074/jbc.RA117.001618 29599293 PMC5995506

[B37] Ma J. Sun M. Dong W. Pan Z. Lu C. Yao H. (2017). PAAR-Rhs proteins harbor various C-terminal toxins to diversify the antibacterial pathways of type VI secretion systems. *Environ. Microbiol.* 19 345–360. 10.1111/1462-2920.13621 27871130

[B38] Ma L.-S. Hachani A. Lin J.-S. Filloux A. Lai E.-M. (2014). *Agrobacterium tumefaciens* deploys a superfamily of type VI secretion DNase effectors as weapons for interbacterial competition in planta. *Cell Host Microbe* 16 94–104. 10.1016/j.chom.2014.06.002 24981331 PMC4096383

[B39] Mougous J. D. Gifford C. A. Ramsdell T. L. Mekalanos J. J. (2007). Threonine phosphorylation post-translationally regulates protein secretion in *Pseudomonas aeruginosa*. *Nat. Cell Biol.* 9 797–803. 10.1038/ncb1605 17558395

[B40] Naushad S. Adeolu M. Wong S. Sohail M. Schellhorn H. E. Gupta R. S. (2015). A phylogenomic and molecular marker based taxonomic framework for the order Xanthomonadales: proposal to transfer the families *Algiphilaceae* and *Solimonadaceae* to the order Nevskiales ord. nov. and to create a new family within the order Xanthomonadales. Antonie van Leeuwenhoek. *Int. J. Gen. Mol. Microbiol.* 107 467–485. 10.1007/s10482-014-0344-8 25481407

[B41] Nguyen V. Douzi B. Durand E. Roussel A. Cascales E. Cambillau C. (2018). Towards a complete structural deciphering of type VI secretion system. *Curr. Opin. Struct. Biol.* 49 77–84. 10.1016/j.sbi.2018.01.007 29414515

[B42] Ostrowski A. Cianfanelli F. R. Porter M. Mariano G. Peltier J. Wong J. J. (2018). Killing with proficiency: integrated post-translational regulation of an offensive Type VI secretion system. *PLoS Pathog.* 14:e1007230. 10.1371/journal.ppat.1007230 30052683 PMC6082577

[B43] Planamente S. Salih O. Manoli E. Albesa-Jové D. Freemont P. S. Filloux A. (2016). TssA forms a gp6-like ring attached to the type VI secretion sheath. *EMBO J.* 35 1613–1627. 10.15252/embj.201694024 27288401 PMC4969574

[B44] Rapicavoli J. Ingel B. Blanco-Ulate B. Cantu D. Roper C. (2018). *Xylella fastidiosa*: an examination of a re-emerging plant pathogen. *Mol. Plant Pathol.* 19 786–800. 10.1111/mpp.12585 28742234 PMC6637975

[B45] Russell A. B. Leroux M. Hathazi K. Agnello D. M. Ishikawa T. Wiggins P. A. (2013). Diverse type VI secretion phospholipases are functionally plastic antibacterial effectors. *Nature* 496 508–512. 10.1038/nature12074 23552891 PMC3652678

[B46] Russell A. B. Wexler A. G. Harding B. N. Whitney J. C. Bohn A. J. Goo Y. A. (2014). A Type VI secretion-related pathway in bacteroidetes mediates interbacterial antagonism. *Cell Host Microbe* 16 227–236. 10.1016/j.chom.2014.07.007 25070807 PMC4136423

[B47] Ryan R. P. Vorhölter F. J. Potnis N. Jones J. B. Van Sluys M. A. Bogdanove A. J. (2011). Pathogenomics of *Xanthomonas*: understanding bacterium-plant interactions. *Nat. Rev. Microbiol.* 9 344–355. 10.1038/nrmicro2558 21478901

[B48] Saddler G. Bradbury J. (2005). “Order III. Xanthomonadales ord. nov,” in *Bergey′s Manual of Systematic Bacteriology*. ed Brenner D. J. Austin: Springer 63–122. 10.1007/0-387-28022-7_3

[B49] Sgro G. G. Oka G. U. Souza D. P. Cenens W. Bayer-Santos E. Matsuyama B. Y. (2019). Bacteria-killing type IV secretion systems. *Front. Microbiol.* 10:1078 10.3389/fmicb.2019.01078 PMC6536674 31164878

[B50] Si M. Wang Y. Zhang B. Zhao C. Kang Y. Bai H. (2017a). The type VI secretion system engages a redox-regulated dual-functional heme transporter for zinc acquisition. *Cell Rep.* 20 949–959. 10.1016/j.celrep.2017.06.081 28746878

[B51] Si M. Zhao C. Burkinshaw B. Zhang B. Wei D. Wang Y. (2017b). Manganese scavenging and oxidative stress response mediated by type VI secretion system in *Burkholderia thailandensis*. *Proc. Natl. Acad. Sci. U.S.A.* 114 E2233–E2242. 10.1073/pnas.1614902114 28242693 PMC5358365

[B52] Silverman J. M. Austin L. S. Hsu F. Hicks K. G. Hood R. D. Mougous J. D. (2011). Separate inputs modulate phosphorylation-dependent and -independent type VI secretion activation. *Mol. Microbiol.* 82 1277–1290. 10.1111/j.1365-2958.2011.07889.x 22017253 PMC3590308

[B53] Silverman J. M. Brunet Y. R. Cascales E. Mougous J. D. (2012). Structure and regulation of the type VI secretion system. *Annu. Rev. Microbiol.* 66 453–472. 10.1146/annurev-micro-121809-151619 22746332 PMC3595004

[B54] Souza D. P. Oka G. U. Alvarez-Martinez C. E. Bisson-Filho A. W. Dunger G. Hobeika L. (2015). Bacterial killing via a type IV secretion system. *Nat. Commun.* 6 1–9. 10.1038/ncomms7453 25743609

[B55] Trunk K. Peltier J. Liu Y. Dill B. Walker L. Gow N. (2018). The type VI secretion system deploys antifungal effectors against microbial competitors. *Nat. Microbiol.* 3 920–931. 10.1038/s41564-018-0191-x 30038307 PMC6071859

[B56] Unterweger D. Kostiuk B. Otjengerdes R. Wilton A. Diaz-Satizabal L. Pukatzki S. (2015). Chimeric adaptor proteins translocate diverse type VI secretion system effectors in *Vibrio cholerae*. *EMBO J.* 34 2198–2210. 10.15252/embj.201591163 26194724 PMC4557670

[B57] Wang J. Yang B. Leier A. Marquez-Lago T. Hayashida M. Rocker A. (2018). Bastion6: a bioinformatics approach for accurate prediction of type VI secreted effectors. *Bioinformatics* 34 2546–2555. 10.1093/bioinformatics/bty155 29547915 PMC6061801

[B58] Wang T. Si M. Song Y. Zhu W. Gao F. Wang Y. (2015). Type VI secretion system transports Zn2+ to combat multiple stresses and host immunity. *PLoS Pathog.* 11:e1005020. 10.1371/journal.ppat.1005020 26134274 PMC4489752

[B59] Weber B. Hennon S. Wright M. Scott N. de Berardinis V. Foster L. (2016). Genetic dissection of the type vi secretion system in *Acinetobacter* and identification of a novel peptidoglycan hydrolase. tagx, required for its biogenesis. *MBio* 7 e1253–e1216. 10.1128/mBio.01253-16 27729508 PMC5061870

[B60] Williams K. P. Gillespie J. J. Sobral B. W. S. Nordberg E. K. Snyder E. E. Shallom J. M. (2010). Phylogeny of gamma proteobacteria. *J. Bacteriol.* 192 2305–2314. 10.1128/JB.01480-09 20207755 PMC2863478

[B61] Zoued A. Durand E. Brunet Y. R. Spinelli S. Douzi B. Guzzo M. (2016). Priming and polymerization of a bacterial contractile tail structure. *Nature* 531 59–63. 10.1038/nature17182 26909579

